# Supplementary material for: Evaluating Models of the Ageing BOLD Response
Source: Hum Brain Mapp. 2024 Oct 18;45(15):e70043. doi: 10.1002/hbm.70043 (PMC11487563; doi:10.1002/hbm.70043)
Supplement: Supplementary file 1 — Data S1. Supporting Information. [file HBM-45-e70043-s001.docx]

## Supplementary Material for Evaluating models of the ageing BOLD response

## Henson, R.N., Olszowy, W., Tsvetanov, K.A., Yadav, P.S., Cam-CAN & Zeidman, P.

## Further details about Haemodynamic modelling (HDM)

Equations 1-8 in the main paper summarise the HDM. Here we expand on its estimation, priors and parameter effects.

Within the model, latent or hidden variables are divided into two types: *states,* which change over time, and *parameters*, which are constant. Only the parameters are estimated from the data, using a Bayesian modelling scheme (Variational Laplace), and the parameters determine the evolution of the states over time.

For the main HDM3 model in the paper, we allowed three parameters to be free: one to capture the magnitude of neural activity, β; one to capture neurovascular effects, namely the rate of decay of vasoactive signal, $\kappa$; and one to capture vascular effects, namely the transit rate of blood flow, 1/$\tau_{h}$. The remaining parameters were fixed at their default priors, for reasons expanded below.

- An important vascular parameter is vessel stiffness (the windkessel effect), which is characterised by Grubb’s exponent $\alpha$ (Grubb et al., 1974). In the range estimated here, increasing $\alpha$ primarily decreases the amplitude of the BOLD response (as well as delay its peak somewhat; see Supplementary Figure S1). Thus its primary effect on the HRF is inversely related to that of the neural efficacy parameter, β, resulting in a positive correlation between their parameter estimates (see Supplementary Figure S2; in general, parameters whose effects are negatively correlated tend to have estimates that are positively correlated, and vice versa). Therefore, we decided to fix the value of $\alpha$ here at 0.33 in the HDM3 model, consistent with previous studies (Friston et al., 2000) (though allowed it to vary in the HDM4 model below). This value of 0.33 is consistent with previous human and animal studies, in which values for $\alpha$ typically range between 0.2 and 0.4 (as reviewed by Leung et al., 2008). Nonetheless, this parameter is likely to change with age. In rats, $\alpha$ has been found to drop from around 0.35 in young individuals (4-5 months) to around 0.25 in very old age (40-41 months) (Dubeau et al., 2011). Thus, it should be kept in mind that some effects of age, particular on the neural parameter $\beta$, could also be accounted for by differences in vessel stiffness. Then again, our finding using the HDM3 model that there are no effects of age on neural efficacy $\beta$ in any ROI except rMC, is unlikely to be explained by $\alpha$, because vessel stiffness is unlikely to differ dramatically between contralateral ROIs like lMC and rMC (see Discussion of main paper).
- The neurovascular parameter $\gamma$ is the rate constant controlling the feedback from blood flow. Together with the decay parameter $\kappa$, the two produce a damped oscillation of the vasodilatory signal. However the primary effect of decreasing $\gamma$ is to delay the HRF, which is similar to the primary effect of decreasing the haemodynamic transit rate 1/$\tau_{h}$ (Supplementary Figure S1), resulting in a high negative correlation between them (Supplementary Figure S2). For the HDM3 model, we therefore fixed $\gamma$ here based on its prior expected value of 0.41Hz from Friston et al. (2000) (though allowed it to vary in the HDM5 model below). This does mean, however, that changes in neurovascular feedback could also reflect changes in haemodynamic transit rate.
- Resting oxygen extraction fraction $E_{0}$ refers to the percentage of the oxygen removed from the blood by tissue during its passage through the capillary network, and is typically assigned a fixed value (40%) in the HDM model (Friston et al., 2000). The effect of $E_{0}$ on the magnitude of the HRF is complex: it can produce an initial negative dip with high values, but at least for values below 40%, increases in $E_{0}$ tend to increase the amplitude of the peak of the HRF (Supplementary Figure S1). This can explain why the present empirical estimates of $E_{0}$ had a high negative correlation with estimates of the neural efficacy parameter, β (Supplementary Figure S2). While evidence suggests that $E_{0}$ increases by about 0.1% per year of adult life (Peng et al., 2014; Leenders et al., 1990), simulations of such changes of up to 7% around the prior of 40% (given the 70 year span in the current sample) showed little effect on the HRF. Therefore we decided to keep $E_{0}$fixed at 40% in the HDM3 model (though allowed it to vary in the HDM6 model below).
- Venous blood volume fraction $V_{0}$ is the proportion of tissue occupied by venous blood, and is also typically assigned a fixed value (4%) in the HDM model (Friston et al., 2000). For grey matter containing small vessels, $V_{0}$ is generally taken to be in the range 1-4% (Buxton et al., 1998; Havlicek et al., 2015; Hua et al., 2019). Leenders et al. (1990) investigated effects of ageing on $V_{0}$, and found it decreases by around 0.05% per year of adult life: from 6.48% for 18 year olds to 3.25% for 88 year olds. Because $V_{0}$ simply scales the BOLD response (see Eq. 5), it is perfectly correlated with the neural efficacy $\beta$, i.e., one cannot estimate both $V_{0}$ and $\beta$ using fMRI data alone. Here we chose to estimate $\beta$ instead, but it should be kept in mind that any effects of ageing on $\beta$ could be accounted for by $V_{0}$ (though again, our finding of a negative BOLD response in rMC cannot be explained soley by $V_{0}$, since $V_{0}$ is always positive).
- We adjusted the values of parameters $\epsilon_{h},$ $r_{0}$ and $\vartheta_{0}$ to correspond to the MRI field strength for the CamCAN data, where $B_{0}=3T$. In previous implementations of this model, the ratio of intra-vascular to extra-vascular signal, $\epsilon_{h},$ was estimated from the data, reflecting uncertainty about its value in the literature (Stephan et al., 2007). Here, we decided to fix its value for stability, as large values of $\epsilon_{h}$ can induce phase transitions that give rise to unrealistic BOLD responses. Following Havlicek et al., 2015, we used the expected value of $\epsilon_{h}=0.44,$ based on the ranges of T2* relaxation rates (for further detail, see the MATLAB script *Heinzle_epsilon_derivation.m* in the HDM Toolbox, <https://github.com/pzeidman/HDM-toolbox>). We set the value of $r_{0}=110s^{-1}$ and $\vartheta_{0}=28.265B_{0}=31.27s^{-1}$, following Heinzle et al. (2016), who drew on the results of Uludag et al. (2009).


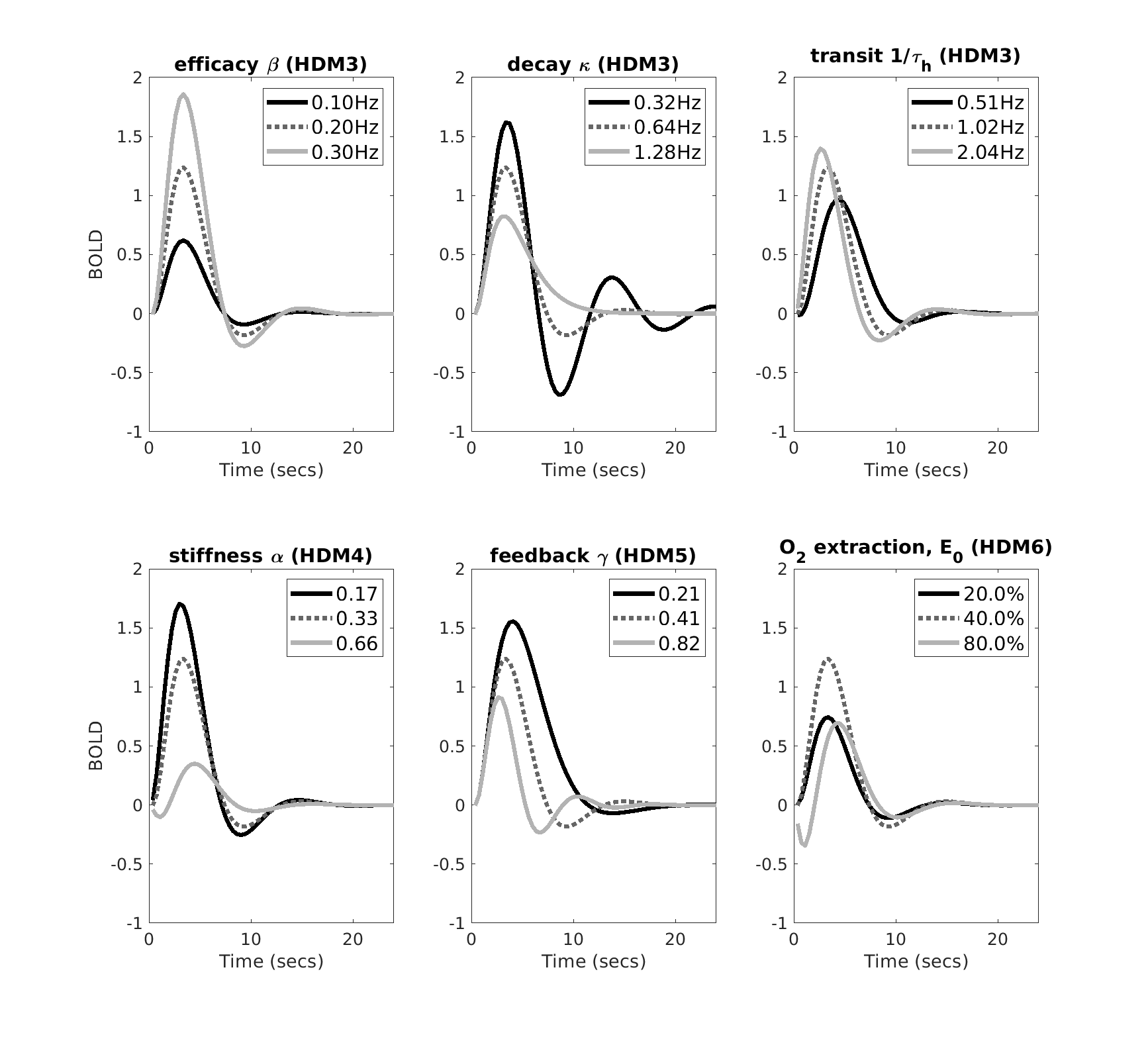


Supplementary Figure S1. Each plot shows the predicted BOLD response under different values of a parameter, indicated by the title. The central parameter value (producing the dotted line) reflects the prior expectation; the dark solid line and light solid line reflect smaller or larger values respectively (the values of all parameters other than the one varied in a plot were fixed at their prior expectation, except neural efficacy, β, which was set to 0.2).


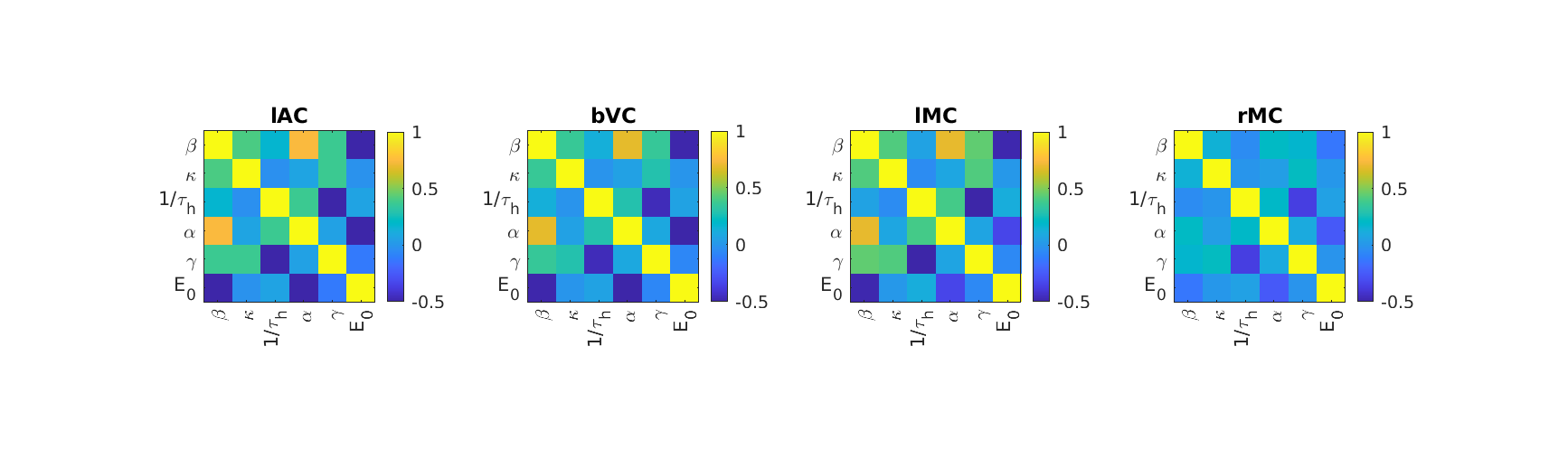


Supplementary Figure S2. The mean across participants of the posterior correlation between HDM parameters after fitting a 6-parameter HDM model (HDM6) to the data in the paper. Apart from rMC, note that the other ROIs have a large positive correlation between neural efficacy $\beta$ and vascular stiffness $\alpha$, and large negative correlations between neurovascular feedback $\gamma$ and haemodynamic transit rate 1/$\tau_{h}$, and between oxygen extraction $E_{0}$and $\beta$.

A summary of the prior expected value and variance for the three free parameters in the main HDM3 model used in the paper are shown in Supplementary Table 1. Note that, in order to enforce positivity constraints on two of these parameters, new parameters $l_{\kappa}$ and $l_{\tau_{h}}$ are introduced, which are log versions of decay rate $\kappa$ and transit rate, $1/\tau_{h}$, respectively. The scaling values of 0.64 and 1.02 are taken from (Friston et al., 2000). The neural efficacy parameter $\beta$ is not constrained to be positive so is untransformed.

Additionally, in order to enforce positivity constraints on the states $f_{in}$, $v$ and $q$, each variable is log-transformed, requiring the equations to be supplemented as follows:

| $\frac{d\ln f_{in}}{dt}=\frac{\dot{f}_{in}}{f_{in}}$ | (9) |
| --- | --- |
| $\frac{d\ln v}{dt}=\frac{\dot{v}}{v}$ | (10) |
| $\frac{d\ln q}{dt}=\frac{\dot{q}}{q}$ | (11) |

**Supplementary Table 1: Free parameters of HDM3 model**

| **Model** | **Parameter** | **Compartment** | **Prior expectation** | **Prior variance** | **Parameterisation** |
| --- | --- | --- | --- | --- | --- |
| HDM3 | $\beta$ | Neural | 0 | 1 | - |
| HDM3 | $l_{\kappa}$ | Neurovascular (CBF) | 0 | 1/32 | $\kappa=0.64s^{-1}\cdot\exp\left( l_{k} \right)$ |
| HDM3 | $l_{\tau_{h}}$ | Vascular  (CBV) | 0 | 1/32 | $\frac{1}{\tau_{h}}=1.02s^{-1}\cdot\exp\left( l_{\tau_{h}} \right)$ |

The HDM models with 4, 5 and 6 free parameters (used later in Sup Mat) included the values in Supplementary Table 1, plus the one or more of rows of Supplementary Table 2.

**Supplementary Table 2: Additional free parameters of HDM4, HDM5 and HDM6 models**

| **Model** | **Parameter** | **Compartment** | **Prior expectation** | **Prior variance** | **Parameterisation** |
| --- | --- | --- | --- | --- | --- |
| HDM4 | $l_{\alpha}$ | Vascular | 0 | 1/32 | $\alpha=0.33\cdot\exp\left( l_{\alpha} \right)$ |
| HDM5 | $l_{\gamma}$ | Neurovascular | 0 | 1/32 | $\gamma=0.41s^{-1}\cdot\exp\left( l_{\gamma} \right)$ |
| HDM6 | $l_{E_{0}}$ | Vascular | 0 | 1/32 | $E_{0}=0.40\cdot\exp\left( l_{E_{0}} \right)$ |

### Group-level model using Parametric Empirical Bayes (PEB)

For the PEB estimation of a group-level model (Zeidman et al., 2019), parameter estimates from all $n$ participants are concatenated into a vector of random variables, $\theta^{(1)}=\left( \theta_{1},\theta_{2}\ldots\theta_{n} \right)$. Between-participant effects were then modelled using a general linear model:

| $\theta^{(1)}=X\theta^{\left( 2 \right)} +\epsilon$ | (12) |
| --- | --- |

The design matrix $X$ included regressors for the effect of each of two covariates (mean over participants and age) on each of $P=3$ haemodynamic parameters ($\beta,l_{\kappa},l_{\tau_{h}}$). This design matrix can be written formally as:

| $X=X_{B}\bigotimes I_{P}$ | (13) |
| --- | --- |

where between-participants design matrix $X_{B}$ contained two columns: a column of ones (to model the average haemodynamic parameters over participants) and the z-scored age of each participant. This was replicated over the three parameters by taking the Kronecker tensor product $\bigotimes$ with the identity matrix of dimension $P$.

Unexplained between-participants variability $\epsilon$ was modelled using a covariance component model, with a separate I.I.D. precision component for each of the three haemodynamic parameters, allowing each type of parameter to have a separate level of between-participants variability.

The parameters $\theta^{\left( 2 \right)}$ and free energy $F$ of the PEB model were estimated using standard routines in the SPM software. We then tested the evidence for the presence versus absence of each covariate on each haemodynamic parameter using an automated procedure. This was an automatic search that iteratively pruned mixtures of group-level parameters $\theta^{\left( 2 \right)}$ from the PEB model, where doing so did not reduce the free energy. This was performed using an analytic approach called Bayesian Model Reduction (Friston et al., 2016).


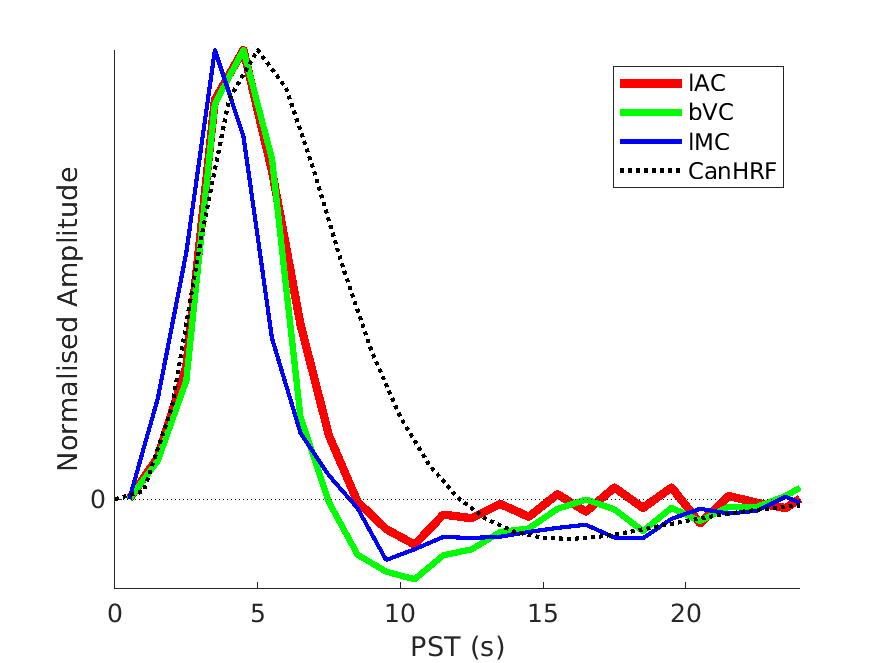


Supplementary Figure S3. Mean across all participants of the FIR fits for lAC, bVC and lMC (solid lines), along with SPM’s canonical HRF (dotted line). The rMC is not shown because it varied so much with age (see main text). Peak amplitude is matched by scaling by the maximum positive value. The lAC and bVC data are from the stimulus-locked model, while lMC is from the response-locked model.

SPM’s canonical HRF is more dispersed than the FIR fits (delayed peak and undershoot). This may not have been noticed before because early studies generating this canonical shape (Friston et al., 2000) did not allow for slice-timing delays. So a delay of TR/2 (typically ~1s) could partly explain this mismatch. Here we did correct the data for different slice-times and were careful to synchronise the GLM (for FIR32 and Can3 basis sets) with the reference slice used for slice-timing. Grinwald et al. (2017) still observed deviations despite slice-timing, though ones that derivatives should be able to accommodate.


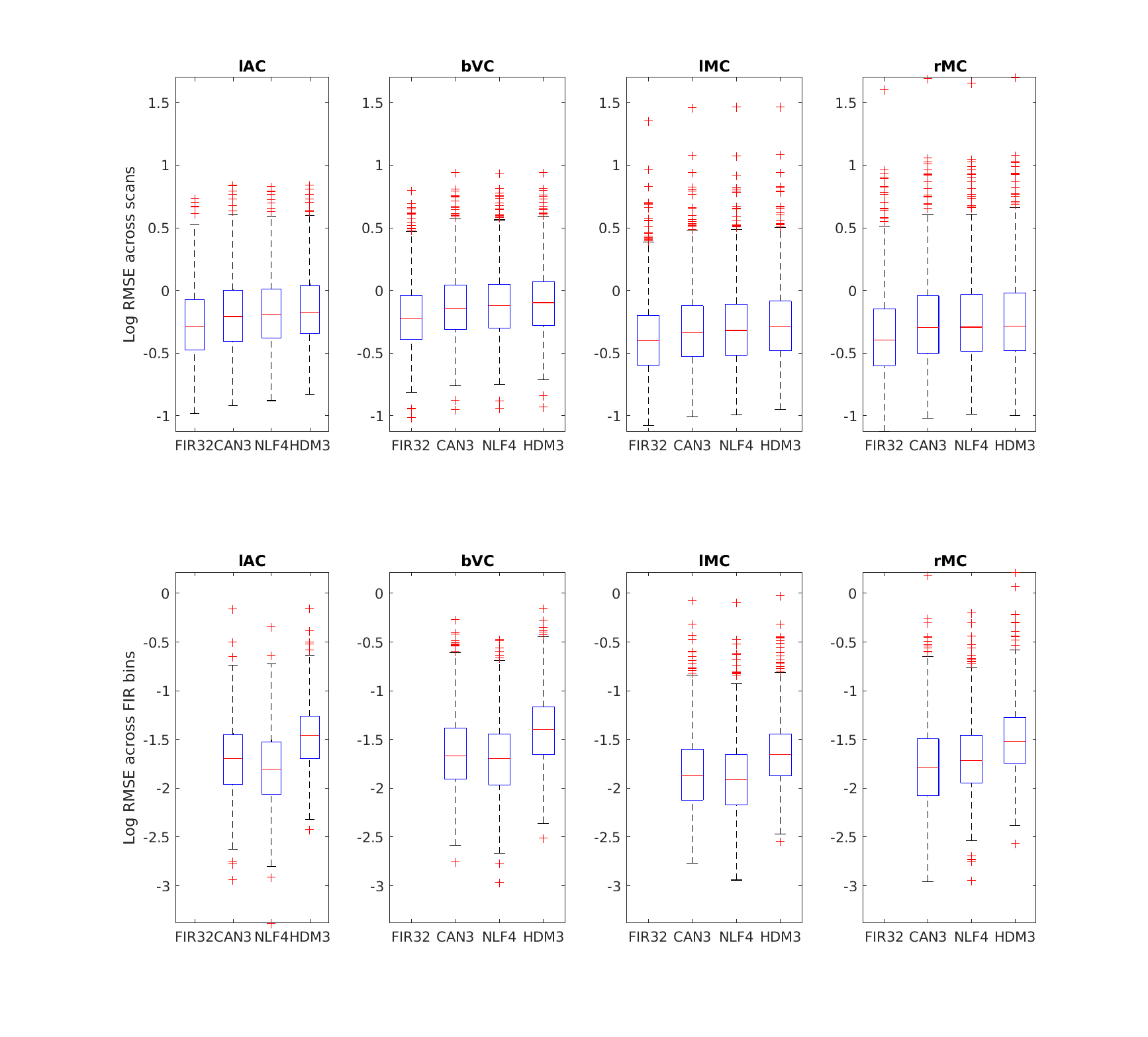


Supplementary Figure S4a. Boxplots (of log) of Root of Mean of Squared Error (RMSE) across scans (top row) or across FIR bins (bottom row) for each ROI and model. Note there is statistical circularity for the FIR model, since the same model was used to define the ROIs in the first place, but its inclusion here at least provides a lower bound, albeit biased, with which to compare the other models. Note also that there can be no RMSE for the FIR32 model in bottom row, and in the top row, the RMSE across scans for the NLF model was calculated by re-inserting the participant- and ROI-specific fitted HRF into each participant’s first-level GLM (hence only 1 effective degree of freedom). The lAC and bVC data are from the stimulus-locked model, while lMC and rMC are from the response-locked model. Note that these measures of model fit ignore differences in model complexity, and so are not as good for generalisation to new data as the cross-validated error in Figure 9.


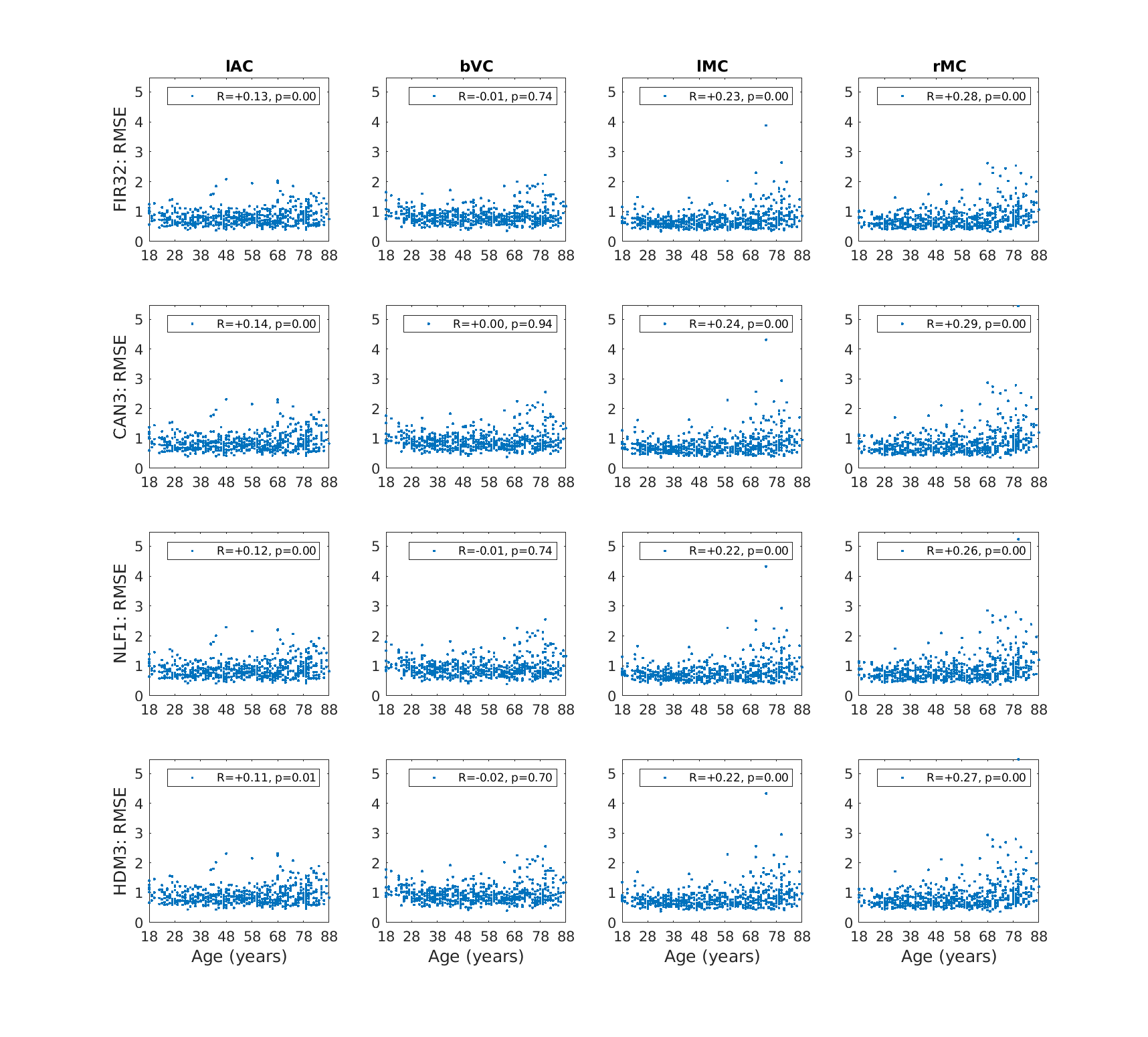


Supplementary Figure S4b. Square-root of Mean of Squared residuals (RMSE) across scans for each model and for each ROI, as a function of participant’s age, together with Spearman correlation R- and p-values. The lAC and bVC data are from the stimulus-locked model, while lMC and rMC are from the response-locked model. Note that the RMSE for the NLF model was calculated by re-inserting the participant- and ROI-specific fitted HRF into each participant’s first-level GLM (hence only 1 effective degree of freedom).


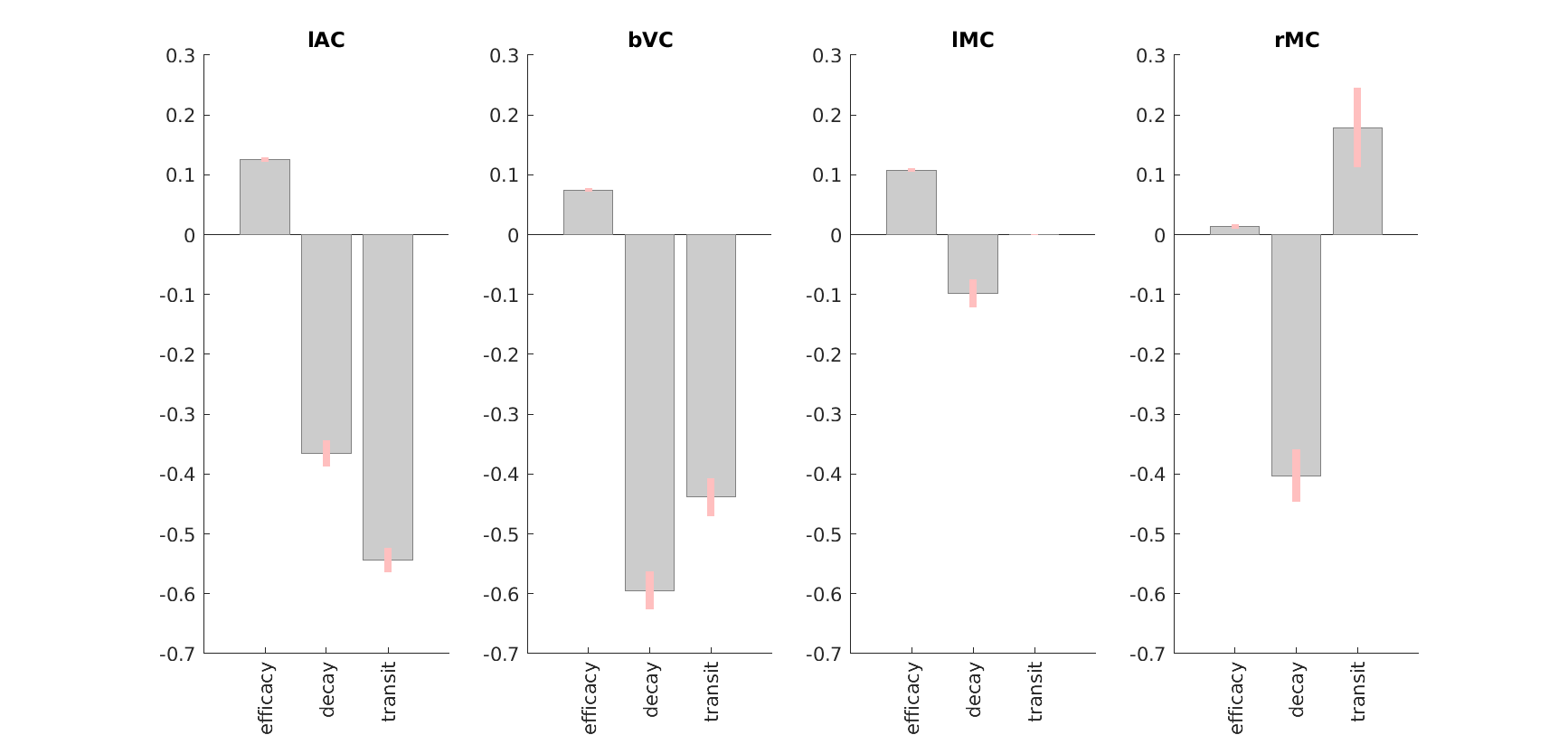


Supplementary Figure S5. Results of PEB BMR on HDM parameters for each ROI for the mean across participants, in terms of deviation of each parameter from its prior expectation. Grey bars show posterior expectation with 90% credible interval in pink; missing bars are parameters that BMR has removed as unnecessary (in terms of maximising evidence for PEB model). For decay and transit parameters, units are log deviations.


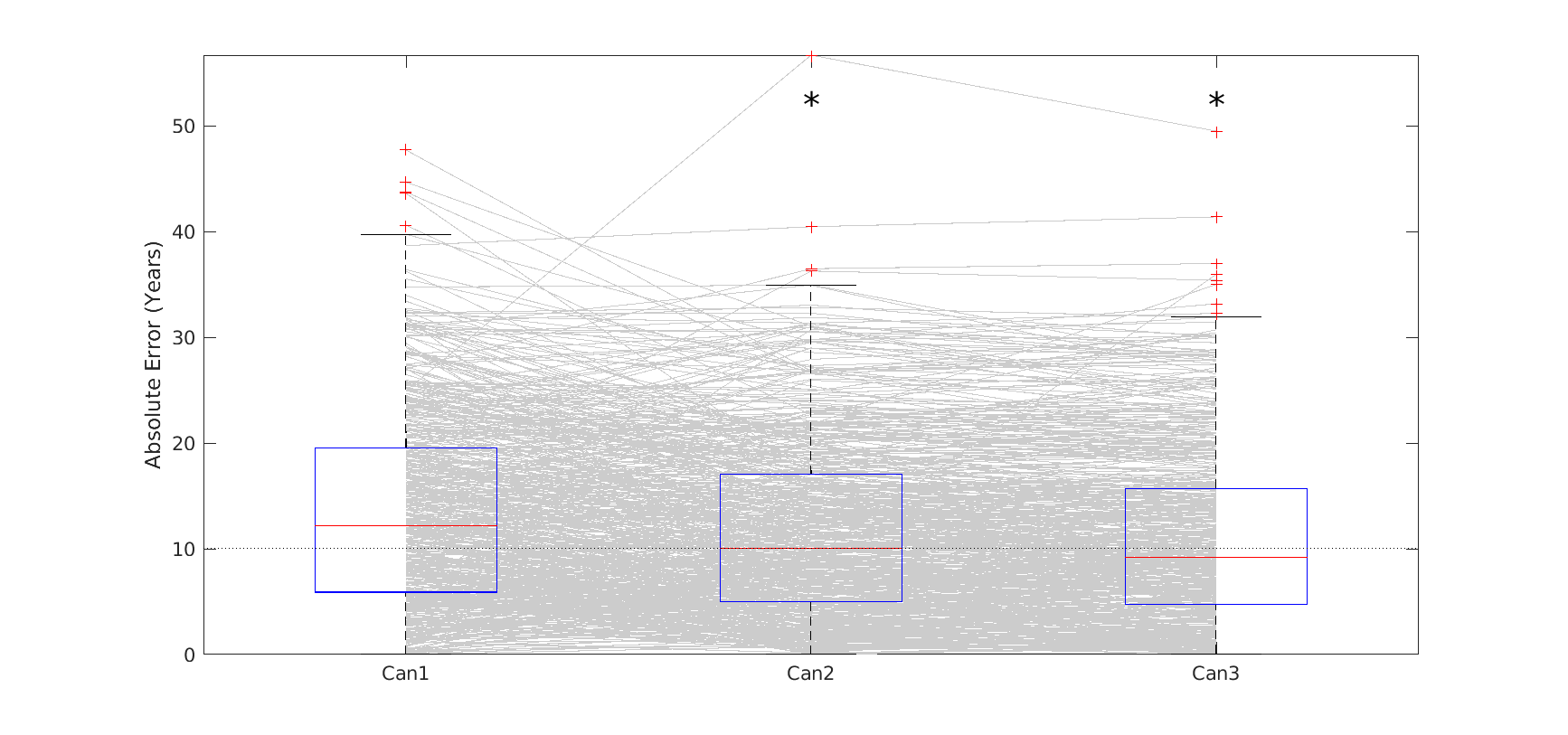


Supplementary Figure S6. Cross-validated absolute error in predicting age, using Can model parameters combined across all ROIs, as the number of basis functions increases, from canonical HRF only (Can1), to adding its temporal derivative (Can2) and then also its dispersion derivative (Can3). Each grey line corresponds to one participant. Superimposed on these lines are boxplots together with outliers (red crosses). An asterisk means that a sign-test revealed significantly better prediction than the simplest (Can1) model. The median error was 12.2 years (Can1), 10.0 years (Can2) and 9.2 years (Can3).


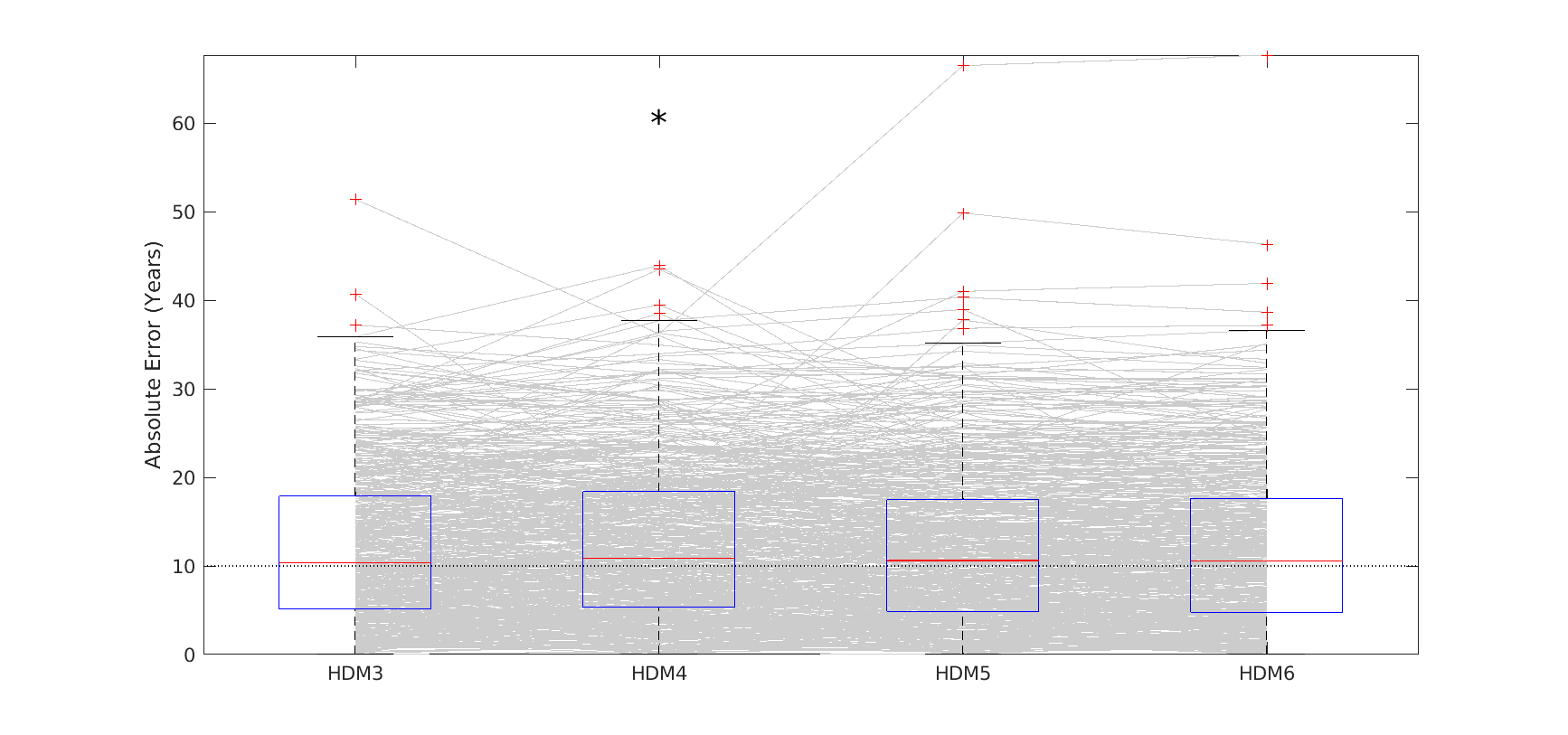


Supplementary Figure S7. Cross-validated absolute error in predicting age, using the HDM model parameters combined across all ROIs, as the number of parameters increased. The HDM3 model is as in main paper; an HDM4 model with the additional vessel stiffness parameter *α*; an HDM5 model with the additional neurovascular feedback parameter *γ*; an HDM6 model with the additional oxygen extraction fraction *E_0_*. Each grey line corresponds to one participant. Superimposed on these lines are boxplots together with outliers (red crosses). An asterisk means that a sign-test revealed significantly different prediction than the simplest (HDM3) model. The average across ROIs of the median error was 10.3 years (HDM3), 10.8 years (HDM4), 10.6 years (HDM5) and 10.5 years (HDM6). Note this is using the parameter estimates before application of PEB.

## References


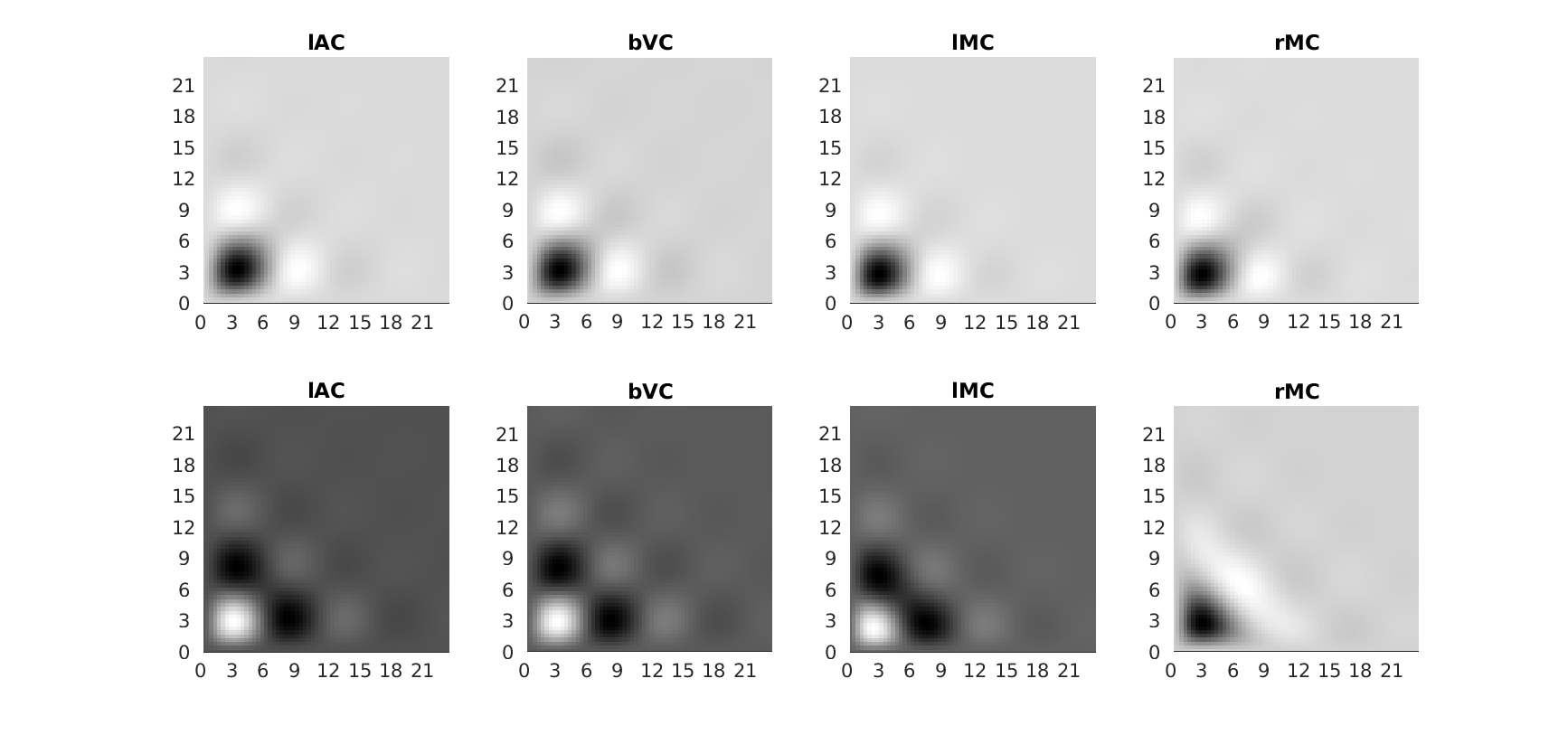


Supplementary Figure S8. Second-order Volterra kernels for each ROI from the HDM3 model, capturing nonlinear effects of time between trial onsets (SOA; plotted on both axes). The top row shows the mean across participants, while the bottom row shows the effect of age. Note that in order to see effects (e.g, in rMC), the grayscale is optimised for each ROI and kernel separately (units are arbitrary).

The dark region in the top row indicates under-additivity (saturation) for SOAs below ~6s in all ROIs, while the white region shows super-additivity for SOAs from ~6s to ~10s, replicating previous findings from Friston et al. (1998). The maximal saturation varied for SOAs 2.6s-3.8s across ROIs, and was highly significant in all ROIs (T(636)>19.6, p<1e-16, though uncorrected for multiple comparisons).

The bottom row shows that the under-additivity below ~6s, as well as the super-additivity between 6-10s, is attenuated with age in all ROIs except rMC, where they are augmented with age. Taking the SOAs with the maximal effect in the top row (which does not bias the orthogonal age effect), the age-related effect was significant for all ROIs, two-tailed T(636)>2.52, p<.05.

The percentage of variance from the two kernels that was explained by the second-order kernel had a median value across participants of 29.2%, 15.6%, 23.9% and 4.5% for lAC, bVC, lMC and rMC respectively. The proportion of that second-order variance related to age (linear and/or quadratic) was 12.1%, 6.1%, 4.5% and 1.3% respectively.

Buxton RB, Wong EC and Frank LR (1998) Dynamics of blood flow and oxygenation changes during brain activation: The balloon model. *Magnetic Resonance in Medicine* 39(6): 855–864. DOI: 10.1002/mrm.1910390602.

Dubeau S, Ferland G, Gaudreau P, et al. (2011) Cerebrovascular hemodynamic correlates of aging in the Lou / c rat : A model of healthy aging. *NeuroImage* 56(4). Elsevier Inc.: 1892–1901. DOI: 10.1016/j.neuroimage.2011.03.076.

Friston KJ, Mechelli A, Turner R, et al. (2000) Nonlinear Responses in fMRI: The Balloon Model, Volterra Kernels, and Other Hemodynamics. *NeuroImage* 12(4). Academic Press: 466–477. DOI: 10.1006/NIMG.2000.0630.

Friston KJ, Litvak V, Oswal A, et al. (2016) Bayesian model reduction and empirical Bayes for group (DCM) studies. *NeuroImage* 128. The Authors: 413–431. DOI: 10.1016/j.neuroimage.2015.11.015.

Grubb RL, Raichle ME, Eichling JO, et al. (1974) The Effects of Changes in PaCO2 Cerebral Blood Volume, Blood Flow, and Vascular Mean Transit Time. *Stroke* 5(5). Lippincott Williams & Wilkins: 630–639. DOI: 10.1161/01.STR.5.5.630.

Havlicek M, Roebroeck A, Friston K, et al. (2015) Physiologically informed dynamic causal modeling of fMRI data. *NeuroImage* 122. Elsevier B.V.: 355–372. DOI: 10.1016/j.neuroimage.2015.07.078.

Heinzle J, Koopmans PJ, den Ouden HEM, et al. (2016) A hemodynamic model for layered BOLD signals. *NeuroImage* 125. Elsevier Inc.: 556–570. DOI: 10.1016/j.neuroimage.2015.10.025.

Hua J, Liu P, Kim T, et al. (2019) MRI techniques to measure arterial and venous cerebral blood volume. *NeuroImage* 187. Neuroimage: 17–31. DOI: 10.1016/J.NEUROIMAGE.2018.02.027.

Leenders KL, Perani D, Lammertsma AA, et al. (1990) Cerebral blood flow, blood volume and oxygen utilization. Normal values and effect of age. *Brain : a journal of neurology* 113 ( Pt 1)(1). Brain: 27–47. DOI: 10.1093/BRAIN/113.1.27.

Leung TS, Tachtsidis I, Tisdall MM, et al. (2008) Estimating a modified Grubb’s exponent in healthy human brains with near infrared spectroscopy and transcranial Doppler. *Physiological Measurement* 30(1). IOP Publishing: 1. DOI: 10.1088/0967-3334/30/1/001.

Peng SL, Dumas JA, Park DC, et al. (2014) Age-related increase of resting metabolic rate in the human brain. *NeuroImage* 98. Neuroimage: 176–183. DOI: 10.1016/J.NEUROIMAGE.2014.04.078.

Stephan KE, Weiskopf N, Drysdale PM, et al. (2007) Comparing hemodynamic models with DCM. *NeuroImage* 38(3). Neuroimage: 387–401. DOI: 10.1016/J.NEUROIMAGE.2007.07.040.

Uludag K, Müller-bierl B and Kâmil U (2009) An integrative model for neuronal activity-induced signal changes for gradient and spin echo functional imaging. *NeuroImage* 48: 150–165. DOI: 10.1016/j.neuroimage.2009.05.051.

Zeidman P, Jafarian A, Seghier ML, et al. (2019) A guide to group effective connectivity analysis, part 2: Second level analysis with PEB. *NeuroImage* 200. Academic Press: 12–25. DOI: 10.1016/J.NEUROIMAGE.2019.06.032.
